# Parallel attentional facilitation of features and objects in early visual cortex

**DOI:** 10.1111/psyp.13498

**Published:** 2019-11-06

**Authors:** Nika Adamian, Søren K. Andersen, Steven A. Hillyard

**Affiliations:** ^1^ School of Psychology University of Aberdeen Aberdeen UK; ^2^ Department of Neurosciences University of California San Diego California; ^3^ Leibniz Institute for Neurobiology Magdeburg Germany

**Keywords:** attention, content/topics, EEG, feature‐based attention, methods, object‐based attention, steady‐state visual evoked potentials

## Abstract

Selective attention can enhance the processing of attended features across the entire visual field. Attention also spreads within objects, enhancing all internal locations and task‐irrelevant features of selected objects. Here, we examine the extent to which attentional enhancement of a feature spreads across attended and unattended objects. Two fully overlapping counter‐rotating bicolored surfaces of light and dark random dots were presented on a gray background of intermediate luminance. This stimulus creates a percept of two separate semitransparent surfaces and allows the measurement of feature‐ and object‐based selections while controlling spatial attention. On each trial, human participants attended to a subset of dots defined by feature (luminance polarity) and object (surface) in order to detect brief episodes of radial motion while ignoring any events in the unattended groups of dots. Attentional selection was assessed by means of steady‐state visual evoked potentials (SSVEPs) and behavioral measures. SSVEP amplitudes recorded at medial occipital electrode sites were modulated both by surface‐based and luminance polarity‐based selection in a manner consistent with independent multiplicative enhancement of attentional effects in different dimensions in early visual cortex. This finding supports the view that feature‐based attention spreads across object boundaries, at least at an early stage of processing. However, SSVEPs elicited at more lateral electrode sites showed a hierarchical pattern of selection, potentially reflecting the binding of surface‐defining features with luminance features to enable surface‐based attention.

## Abstract 2

Selective attention enhances the processing of attended features across the visual field as well as all the features of attended objects. The current study tested how feature‐based attention interacts with object‐based attention. Steady‐state visual evoked potentials showed that at an early stage of processing feature‐based attention spreads across object boundaries.

## 1 INTRODUCTION

The visual system has a limited processing capacity and is continuously bombarded with sensory stimulation. Consequently, prioritizing and selection based on current goals is crucial for adaptive behavior. Early studies of selective attention focused primarily on spatial selection or the attentional “spotlight,” which enhances processing of locations where relevant information is expected (e.g., Posner, Snyder, & Davidson, 1980). However, cues for attentional selection can also be nonspatial, including features (Hayden & Gallant, 2009; Hillyard & Münte, 1984; Martinez‐Trujillo & Treue, 2004; McAdams & Maunsell, 2000), objects (Blaser, Pylyshyn, & Holcombe, 2000; Duncan, 1984; Houtkamp, Spekreijse, & Roelfsema, 2003; Serences, Schwarzbach, Courtney, Golay, & Yantis, 2004; Yantis, 1992), and temporal intervals (Coull & Nobre, 1998; Doherty, 2005; Ghose & Maunsell, 2002).

Feature‐based attention prioritizes perceptual processing of stimuli that contain a task‐relevant feature such as a specific color, orientation, or direction of motion, or a combination of such features (Baldassi & Verghese, 2005; Hayden & Gallant, 2009; Martinez‐Trujillo & Treue, 2004; Reynolds & Chelazzi, 2004). Importantly, feature‐based selection typically spreads across the visual field even to the irrelevant or to‐be‐ignored locations (Andersen, Hillyard, & Müller, 2013; Bartsch, Donohue, Strumpf, Schoenfeld, & Hopf, 2018; Liu & Mance, 2011; Rossi & Paradiso, 1995; Sàenz, Buraĉas, & Boynton, 2002). This global selection can benefit certain tasks such as visual search (Treisman, 1988; Wolfe, 1994) by restricting the search to a subset of objects containing the attended feature, or it can impede performance in cases when the task‐relevant feature differs across locations (Andersen et al., 2013; Sàenz, Buraĉas, & Boynton, 2003).

In most visual scenes, features are not only organized in space but are also perceptually grouped based on connectedness or one or more Gestalt principles (Goldsmith, 1998; Kimchi, Yeshurun, & Cohen‐Savransky, 2007). In the strongest case, this connectedness leads to formation of a coherent object, which can be selected as a whole, with all its properties and parts preferentially processed (Scholl, 2001). The consequences of object‐based selection are twofold. On the one hand, attention automatically spreads inside the object, both in space (Egly, Driver, & Rafal, 1994; Martínez, Teder‐Salejarvi, & Hillyard, 2007; Martínez et al., 2006) and in the feature domain (Katzner, Busse, & Treue, 2009; O'Craven, Downing, & Kanwisher, 1999; Schoenfeld et al., 2003; Valdes‐Sosa, Bobes, Rodriguez, & Pinilla, 1998; Wannig, Rodríguez, & Freiwald, 2007). On the other hand, attention is constrained by the object boundaries such that there is a cost to attending two separate objects independently of the number of attended features (Baylis & Driver, 1993). Thus, object boundaries guide the coselection of irrelevant features belonging to the attended object and may restrict the enhancement of those features outside the attended region of the visual field (Boehler, Schoenfeld, Heinze, & Hopf, 2011; Boynton, Ciaramitaro, & Arman, 2006). However, the extent to which object boundaries impede otherwise global selection of features remains unclear.

One of the most influential experimental paradigms in the studies of object‐based attention uses superimposed rotating dot patterns with attention directed to one of the two transparent surfaces (Khoe, Mitchell, Reynolds, & Hillyard, 2005; Mitchell, Stoner, Fallah, & Reynolds, 2003; Valdes‐Sosa et al., 1998; Valdes‐Sosa, Cobo, & Pinilla, 2000). In this type of experiment, participants are asked to judge brief episodes of translational motion in one of the two dot fields that rotate in opposite directions. The results typically show that translations in the attended surface are reported more accurately than translations in the unattended surface, suggesting that the cued perceptual surface is preferentially processed. This form of surface‐based attention requires binding of continuously moving elements into a cohesive object and rules out the possibility of selection by space (surfaces are superimposed) or by a singular feature (direction of target motion is unpredictable).

Evidence from psychophysical studies points to the object basis of surface selection. Stoner and Blanc (2010) probed the tuning of object‐based cues by switching features or other properties of the rotating dot fields in a psychophysical experiment. They found that cueing was specific to the dots making up the cued surface rather than the features of the surface, suggesting that surfaces can be a unit of selection on their own. Moreover, Festman and Braun (2010, 2012) found that the spread of surface‐based attention conforms to the global motion flow rather than following linear motion direction, suggesting that moving surfaces are treated like integrated objects and not as instances of local motion. Overall, these studies support the view that surface‐based selection is a (perhaps primitive) form of object‐based selection, and thus surface boundaries might constrain the spread of purely feature‐based attention.

An alternative interpretation would consider surface‐based attention to be an extension of feature‐based selection. Surfaces are comprised of spatially bound items coselected based on a common feature such as direction of motion and/or color. Previous studies exploring simultaneous selection of multiple features, including space, color, and orientation, found that features within a conjunction are selected independently and in parallel (Andersen, Fuchs, & Müller, 2011; Andersen, Hillyard, & Müller, 2008; Andersen, Muller, & Hillyard, 2015). If the same principle applies to transparent surfaces, selection of a surface would be based on independent selection of its separate features, and such feature selection would be global across surfaces.

The present study used a version of the well‐studied paradigm based on superimposed transparent surfaces (Valdes‐Sosa et al., 1998, 2000; Wannig et al., 2007), incorporating luminance polarity as an additional, independent feature. We presented observers with two spatially superimposed surfaces rotating in opposite directions. Both surfaces consisted of random arrays of light and dark dots. On each trial, participants attended either dark or light dots on either one of the two surfaces in order to detect brief radial motion targets. Frequency‐tagged steady‐state visual evoked potentials (SSVEPs) elicited by each of the four types of dots were recorded concurrently. If feature‐based attentional selection is not constrained by object boundaries, feature‐based enhancement of the attended dots should be extended to the dots of the attended luminance belonging to the unattended surface. Alternatively, if such constraints exist, the transfer of feature‐based attentional enhancement from one surface to the other should be impeded.

## 2 METHOD

### 2.1 Participants

The study included 15 participants (4 female, 3 left‐handed) aged 18–23 years (*M* = 19.9 ± 1.6), all of whom reported normal or corrected‐to‐normal visual acuity. All participants provided informed written consent, and the study was conducted in accordance with the ethical guidelines of the University of California San Diego. Five additional participants were excluded because of poor performance on the task, excessive EEG artifacts, or technical issues during EEG recording.

### 2.2 Stimuli and procedure

Stimulation was presented in a dimly lit room on a 19‐inch computer monitor with 640 × 480 pixels resolution and a refresh rate of 120 Hz. Stimuli were created using MATLAB (MathWorks Inc., Natick, MA) and the Cogent Graphics package (http://www.vislab.ucl.ac.uk/cogent.php). Participants were seated at a viewing distance of 80 cm.

**Figure 1:**
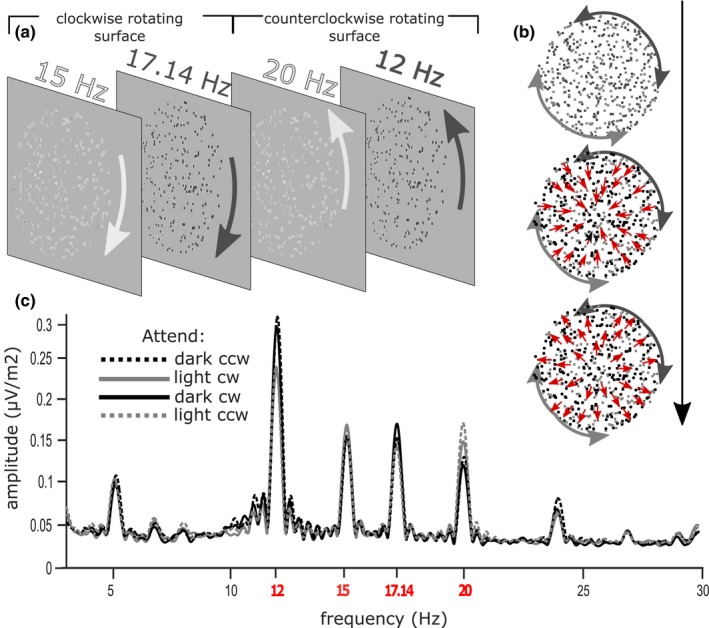
Stimulus display and EEG spectra. (a) Schematic representation of the four components of the stimulus arrays. Four overlapping dot groups defined by luminance and direction of rotation each flickered at a unique frequency and were perceived as two semitransparent surfaces rotating in opposite directions. (b) Schematic representation of the radial motion events: half of the dots of one dot group move radially inward and then back outward while keeping the rotational speed constant (this created a spiraling motion in and out). Arrows were not shown during stimulation. (c) Grand‐averaged amplitude spectra over a broad cluster of temporo‐occipital electrodes obtained by Fourier transformation zero padded to 16,384 points (see SSVEP Recordings and Analysis for details). Stimulation frequencies are labeled in red

Stimuli were presented against a midgray background (20 cd/m^2^). Each trial started with the presentation of a red fixation cross. A circular arrow (light gray or dark gray, directed clockwise or counterclockwise) instructed participants to attend to the corresponding luminance polarity (dark, 5 cd/m^2^ or light, 35 cd/m^2^; ±75% Weber contrast) and direction of rotation of the to‐be‐attended surface. This cue was presented for 600 ms. Immediately after the cue, two superimposed counter‐rotating circular dot patterns (perceived as separate surfaces) consisting of 120 dots each (60 dark and 60 light) were presented for 3,200 ms (see Figure 1). Dot patterns were contained within a circle having a diameter of 13.97° of visual angle and centered on the screen. Each of the four types of dots flickered at an individual frequency synchronized to the refresh rate of the screen: dark counterclockwise (12.00 Hz), light counterclockwise (20.00 Hz), dark clockwise (15.00 Hz), and light clockwise (17.14 Hz). To prevent systematic overlap of dark and light dots, potentially inducing a depth cue, dots were drawn in random order on each frame. To discourage tracking of individual dots, each dot had a limited lifetime (0.5% chance of being redrawn at a new position on each frame).

Both surfaces rotated rigidly around the fixation point with a constant speed of 30 degrees/s except for brief intervals (500 ms) of 50% coherent radial motion. During these motion events, half of the dots of one of the four types would move first centripetally (inward) then centrifugally (outward), resulting in perceptual contraction and expansion. All dots continued rotating at the same speed, thus target and distractor dots retained the object‐defining property. Motion events could occur randomly in either the attended or in one of the unattended dot types. Participants were asked to press a button whenever they detected a motion event in the attended dot type (target) while ignoring motion events of the three unattended types of dots (distractors). The responding hand was changed after half of the trials were completed.

The experiment consisted of 560 trials presented in seven blocks of 80 trials each. Between one and three motion events (either targets or distractors, at random) were presented in 240 randomly distributed trials. The remaining 320 trials did not contain any targets or distractors. The earliest start of the motion event was 500 ms after onset of the rotating and flickering dots, and the onsets of consecutive motion events within a trial were separated by at least 700 ms.

Trials having the four different cueing conditions (attend dark counterclockwise, attend light counterclockwise, attend dark clockwise, attend light clockwise) were presented in random order. Within each of the four cueing conditions, a total of 120 radial motion events were presented, 30 for each type of dot. Thus, for each cueing condition, 25% of the events were targets and 75% were distractors. Feedback about participants' behavioral performance was provided after every block.

### 2.3 Behavioral data analysis

Hits and false alarms were defined as responses delivered in the time window between 300 and 950 ms after targets and distractors, respectively. False alarm rates for the three types of distractors (attended luminance, unattended surface: L+S−, unattended luminance, attended surface: L−S+, and unattended luminance, unattended surface: L−S−) were calculated separately and submitted to a one‐way repeated measures analysis of variance (ANOVA) with Greenhouse‐Geisser correction for nonsphericity.

### 2.4 SSVEP recordings and analysis

Brain electrical activity was recorded at a sampling rate of 250 Hz from 61 Ag/AgCl scalp electrodes using a modified 10‐10 system montage by means of an SA Instrumentation amplifier, with bandpass set at 0.1 to 80 Hz (half amplitude low‐ and high‐frequency cutoffs, respectively). The montage included five additional electrodes located inferior to the occipital row of electrodes (Teder‐Salejarvi, Di Russo, McDonald, & Hillyard, 2005). Impedances were kept below 5 kΩ, and the left earlobe served as an online recording reference. Vertical and horizontal electro‐oculograms were recorded from an additional bipolar montage at the outer canthi of the eyes and another electrode below the right eye referenced offline against Fp1.

EEG data analysis was performed using the EEGLAB toolbox (Delorme & Makeig, 2004) and custom‐built MATLAB scripts (The MathWorks). No offline filters were applied to the data. Analysis epochs were extracted from 600 to 3,100 ms after the onset of rotational motion, including only trials without targets or distractors to ensure that radial motion events did not interfere with the allocation of selective attention. This time range was chosen to exclude the visual evoked responses to the stimulus onset and offset and to allow sufficient time for the SSVEP to build up. The overall mean and linear trend were subtracted from each epoch (detrending). Trials with eye movements larger than 20 µV or blinks were rejected, and the remaining epochs were submitted to an automated preprocessing procedure (Junghöfer, Elbert, Tucker, & Rockstroh, 2000) for further trial exclusion and channel approximation. This procedure replaced artifact‐contaminated sensors with statistically weighted spherical interpolation. The average trial rejection rate was 22.5% (±7.2%) of trials across participants and conditions. The average number of interpolated channels was 2.83 (±1.58%).

Subsequently, trials were subjected to a scalp current density (SCD) transformation using spherical spline interpolation (Pernier, Perrin, & Bertrand, 1988; Perrin, Pernier, Bertrand, & Echallier, 1989). SCDs are reference free, offer higher spatial resolution, and allow for a better correspondence of scalp topographies to the underlying cortical generators (Tenke & Kayser, 2005). SSVEP amplitudes at the four stimulation frequencies (12, 15, 17.14, 20 Hz) were obtained from the SCD‐transformed epochs as the absolute value of the complex Fourier coefficients.

**Figure 2:**
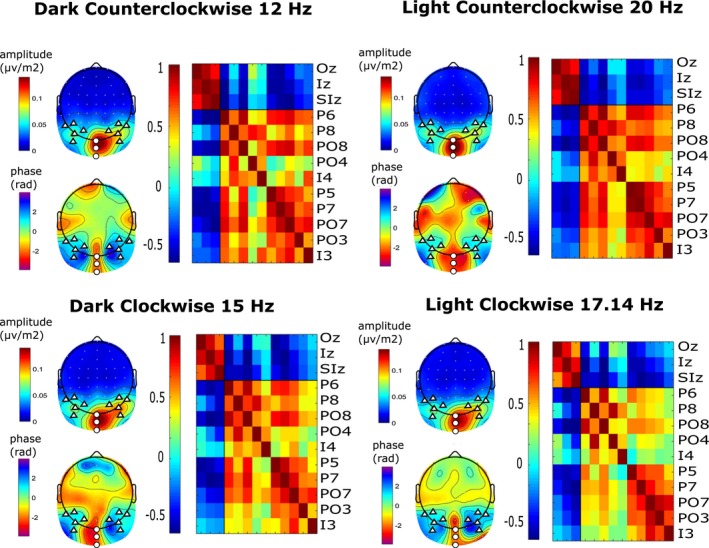
Topographical maps and phase coherence of SSVEPs. For each stimulation frequency: top left: Grand mean scalp current density (SCD) map averaged across conditions. Maximum amplitudes were obtained at midline occipital (white circles) and lateral parieto‐occipital (white triangles) sites. Note that SCD‐transformed data are reference free. Bottom left: Grand mean SSVEP phase map averaged across conditions. Cluster borders were clearly defined by the phase differences. All phases were rotated to align Oz electrodes to minus π/2 radians. Topographies were created using 2D biharmonic spline interpolation (*topoplot* function in EEGLAB). Right: Phase coherence for all pairs of electrodes averaged across participants and conditions. Phase coherence was defined as the cosine of the phase difference between the two electrodes of each pair; that is, a value close to 1 corresponds to an almost identical phase of the two electrodes of the pair for all conditions and subjects. The columns of these phase plots represent the same electrode sites as labeled in the rows, starting with Oz at the left and continuing to I3 at the right

Based on the examination of amplitude and phase of SSVEPs at all electrodes (see Figure 2), we identified two clusters of electrodes for further analysis: midline occipital (Oz, Iz, SIz) and lateral parieto‐occipital (P6, P8, PO4, PO8, I4, P5, P7, PO7, PO3, I3). The selection of electrodes was based on overall signal strength, while grouping of the two clusters was based on phase similarity within clusters and phase dissimilarity across clusters, an approach introduced by Andersen, Muller, and Martinovic (2012). SSVEP amplitudes were averaged over the electrodes in each cluster separately and rescaled by dividing each of the amplitudes by the mean over all attentional conditions (Andersen et al., 2011, 2008, 2013) for each participant and frequency. This resulted in normalization of the SSVEP amplitudes to a mean of 1.0, which allowed subsequent collapsing across frequencies to produce averaged normalized SSVEP amplitudes for the different attentional conditions in each electrode cluster. The resulting collapsed conditions are L+S+ (attended luminance on the attended surface), L+S− (attended luminance on the unattended surface), L−S+ (unattended luminance of the attended surface), and L−S− (unattended luminance on the unattended surface). Across participants, the number of trials used for averaging was 61.3 (range 42‒74) for L+S+ condition, 62.5 (range 48‒78) for L+S− condition, 61.8 (range 43‒80) for L−S+ condition, and 62.12 (range 43‒77) for L−S− condition.

Averaged amplitudes collapsed across the frequencies were submitted to a 2 × 2 repeated measures ANOVA with factors of luminance (L+ vs. L−) and surface (S+ vs. S−), for each cluster separately. Planned analyses additionally included the following contrasts: (a) L−S− versus L−S+ (to estimate the effect of object‐based attention, that is, the spread of attentional facilitation to the unattended feature of the attended surface); (b) L−S− versus L+S− (to estimate the spread of feature‐based attention onto the unattended surface); and (c) L+S− versus L−S+ (to compare the strength of feature‐based and surface‐based facilitation). *P* values were corrected for multiple comparisons using the Bonferroni‐Holm method.

## 3 RESULTS

### 3.1 Behavioral data

The majority of responses were hits, that is, responses to the motion events in the dot type of the attended luminance on the attended surface (L+S+). The average hit rate was 80.9% with a mean reaction time of 628 ms (Table 1). False alarms (responses to the motion events in one of the three unattended dot types) accounted for 8.4% (±7.5%) of all responses. The high average hit rate combined with the low rate of false alarms indicates that participants were well able to perform the task and follow the attentional cues. False alarm rates depended upon distractor type, *F*(2, 14) = 29.15, *p* < 10^–5^, *η* ^2^ = 67.6%. Specifically, participants produced more false alarms to L+S− distractors compared to L−S+ distractors, *t*(14) = 3.76, *p* = .002, *d* = 0.97, and more false alarms to L−S+ distractors compared to L−S− distractors, *t*(14) = 3.82, *p* = .002, *d* = 0.99.

**Table 1:** Behavioral results

Stimulus	Events responded to (%)		Reaction time (ms)	
	*M*	*SD*	*M*	*SD*
L+S+	80.9	7.92	628.4	51.65
L+S−	16.4	7.16		
L−S+	7.2	6.52		
L−S−	1.6	1.4		

Notes

Response rates are reported for each stimulus type: target (L+S+) and distractors (L+S−, L−S+, L+S−, L−S−). For target responses (hits), reaction time is also reported.

### 3.2 SSVEP amplitudes

**Figure 3:**
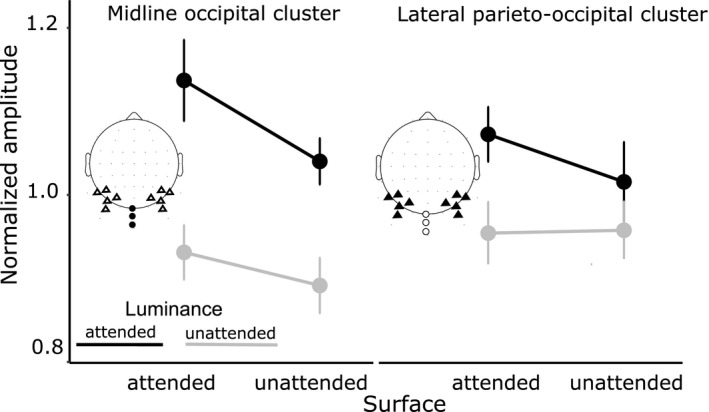
Normalized grand‐averaged SSVEP amplitudes for all attentional conditions in two electrode clusters. Corresponding electrode locations are shown on the scalp maps. Error bars are within‐subject 95% confidence intervals (Morey, 2008)

As shown in Figure 1c, all dot groups elicited clear SSVEPs at their tagging frequencies. Figure 3 shows the summary of normalized and averaged SSVEP amplitudes for each of the four attention conditions. Within the midline occipital cluster, SSVEP amplitudes were significantly enhanced by luminance‐based attention (L+ vs. L−), *F*(1, 14) = 54.44, *p* < 10^–5^, *η* ^2^ = 61.64%, and by surface‐based attention (S+ vs. S−), *F*(1, 14) = 17.31, *p* < 10^–3^, *η* ^2^ = 9.15%. There was also a significant interaction between the two types of attention, *F*(1, 14) = 5.16, *p* = .04, *η* ^2^ = 1.9%. Pairwise comparisons revealed that the unattended luminance was enhanced when it belonged to the attended surface compared to when it belonged to the unattended surface (L−S+ vs. L−S−: *t*(14) = 2.53, *p* = .024, *d* = 0.6), which is a hallmark of object‐based attention. At the same time, attentional enhancement extended to the attended luminance on the unattended surface (L−S− vs. L+S−: *t*(14) = 7.09, *p* < 10^–5^, *d* = 1.8), indicating a global effect of luminance‐based attention.

Within the lateral parieto‐occipital cluster, SSVEP amplitudes were significantly enhanced by luminance‐based (L+ vs. L−), *F*(1, 14) = 11.14, *p* = .005, *η* ^2^ = 30.85%, but not by surface‐based (S+ vs. S−), *F*(1, 14) = 2.4, *p* = .14, *η* ^2^ = 2.8%, attention. The interaction between these two types of attention was also significant, *F*(1, 14) = 6.86, *p* = .02, *η* ^2^ = 3.6%. Pairwise comparisons did not confirm an effect of object‐based selection (L−S− vs. L−S+: *t*(14) = 0.2, *p* = .84, *d* = 0.05) and only a trend toward a global luminance‐based effect (L−S− vs. L+S−: *t*(14) = 1.8, *p* = .09, *d* = 0.4) in this electrode cluster. In both clusters, luminance‐based selection was stronger than surface‐based selection (midline: *t*(14) = −4.29, *p* < 10^–7^, *d* = 1.1; lateral: *t*(14) = −3.09, *p* = .009, *d* = 0.79).

As shown in Figure 2, the lateral parieto‐occipital cluster combines sensors located above the left and right hemispheres as they show enhanced interhemisphere coherence (Figure 2, right panels). We did not observe hemisphere lateralization of feature‐based or surface‐based attention. A 2 × 2 × 2 repeated measures ANOVA with factors subcluster (left or right), luminance (L+ vs. L−), and surface (S+ vs. S−) did not reveal any significant interactions of attention with subcluster (subcluster × luminance: *F*(1, 14) = 2.8, *p* = .11, *η* ^2^ = 2%; subcluster × surface: *F*(1, 14) = 0.19, *p* = .66, *η* ^2^ = 0.06%).

To further explore the nature of the interaction between luminance‐based and surface‐based attention, we tested the additivity of this relationship using logarithmically transformed SSVEP amplitudes. The above‐described significant interaction in raw (normalized) data suggests that the amount of attentional enhancement due to surface‐based selection varies based on the state of feature‐based attention. In the analysis of log‐transformed amplitudes, we are testing the hypothesis that this variability is proportional; that is, each type of attention enhances SSVEP amplitudes by a certain constant percentage. Such proportionality of the attentional modulations would be indicated by the absence of an interaction between feature‐based and surface‐based attention in log‐transformed data.

In the midline occipital cluster, log‐transformed SSVEP amplitudes were significantly modulated by both luminance‐based, *F*(1, 14) = 49.86, *p* < 10^–6^, *η* ^2^ = 62.57%, and surface‐based, *F*(1, 14) = 14.8, *p* =.002, *η* ^2^ = 8.6%, attention. However, the interaction between the two types of attention no longer reached statistical significance, *F*(1, 14) = 3.29, *p* = .09, *η* ^2^ = 1%, indicating a proportional multiplicative interaction in the original, untransformed data. As a result of these multiplicative attention effects, luminance‐based attention enhanced SSVEP amplitudes by approximately 20%, while surfaced‐based attention produced an approximate 7% enhancement.

In the lateral parieto‐occipital cluster, log‐transformed SSVEP amplitudes were modulated by luminance‐based, *F*(1, 14) = 10.7, *p* = .006, *η* ^2^ = 30.11%, but not surface‐based attention, *F*(1, 14) = 2.27, *p* = .15, *η* ^2^ = 2.7%. There was a significant interaction between the two types of attention, *F*(1, 14) = 6.48, *p* = .02, *η* ^2^ = 3.6%, reflecting the fact that the enhancement due to luminance‐based attention was larger for the attended surface (L+S+ vs. L−S+: *t*(14) = 4.64, *p* < 10^–4^, *d* = 1.19) than for the unattended surface (L+S− vs. L−S−: *t*(14) = 1.76, *p* = .1, *d* = 0.45). The presence of this interaction in the log‐transformed data suggests a synergistic effect between the two types of attention resulting in predominant selection of the cued stimulus. Importantly, using log‐transformed data did not change the total amount of variance explained by each of the models, meaning that the removal of the interaction in the midline occipital cluster was not due to a loss in explanatory power. Overall, the analysis of log‐transformed amplitudes confirmed independent multiplicative enhancement of luminance‐based and surface‐based attention in the midline cluster and suggested a hierarchical integration of luminance and surface selections in the lateral cluster.

For the analysis reported above, electrode clusters were selected based on the average topography across all participants. To test the robustness of the results against individual topographical differences, we repeated the analysis excluding the electrodes at the border of the clusters (I3/4, PO7/8, PO3/4). This analysis produced the same pattern of results as the main analysis.

## 4 DISCUSSION

This study set out to test the extent to which object‐based attention restricts the global spread of feature‐based enhancement. Using the task of selecting a surface based on its luminance and direction of motion, we found electrophysiological signatures of both object attention and feature‐based attention. Specifically, while SSVEP amplitudes from occipital sites were largest in response to the attended dots, they were also enhanced to dots of the unattended luminance belonging to the attended surface (indicative of object selection) and to the dots of the attended luminance belonging to the unattended surface (indicative of feature selection). However, we identified two clusters of electrodes with distinctly different patterns of interaction between feature‐based and surface‐based attention.

At the midline occipital cluster of electrodes (Oz, Iz, SIz), attention to luminance enhanced SSVEP amplitudes to the attended dots on both the attended and unattended surfaces, while surface‐based attention enhanced the SSVEP amplitudes to dots of both the attended and unattended luminances, consistent with a multiplicative combination of independent attention effects. Thus, at least for neural pathways contributing to this cluster, the spread of luminance‐based attention was not restricted by surface boundaries. Similar patterns of attentional coselection were previously shown for combinations of space and color (Adamian, Slaustaite, & Andersen, 2019; Andersen et al., 2011; Hayden & Gallant, 2005) and orientation and color (Andersen et al., 2008, 2015). Together, these findings strongly suggest that attentional facilitation is applied independently to the different task‐relevant dimensions or features that define the target stimulus, with the multiplicative effects of attention to each feature summing to produce maximal enhancement of the SSVEP to the target feature combination.

Moreover, in the lateral parieto‐occipital cluster (P6, P8, PO4, PO8, I4, P5, P7, PO7, PO3, I3) attentional enhancement was predominantly focused on the cued stimulus, that is, the dots of the attended luminance on the attended surface, with luminance‐based selection being stronger than surface‐based selection. This pattern matches the behavioral data (most false alarms occur to the stimulus of the attended luminance on the unattended surface). One possible mechanism for this data pattern would be a hierarchical selection, whereby selection of one stimulus attribute or feature is contingent upon selection of the other. In the present case, it appears most likely that selection of surface would be contingent upon the more robust selection of luminance value. A similar hierarchical contingency was observed by Stoner and Blanc (2010), who found that surface‐based selection was spatially restricted to texture elements moving in the cued direction. Evidence for hierarchical selection has also been demonstrated in studies of ERPs to transient stimuli, where the processing of relevant stimulus features was found to be contingent upon their presentation at an attended location (Anllo‐Vento & Hillyard, 1996; Hillyard & Münte, 1984). However, when the location cue was made less salient, this hierarchical contingency was lost. With the timing information available in transient ERP recordings, it was possible to show that selection of the salient location preceded selection of other target‐defining features, but with SSVEPs the order of hierarchical selections could not be established. The selection hierarchy observed here with SSVEPs also differs from the aforementioned studies with transient stimuli in that here spatial selection was not possible, and selection of the object/surface was contingent upon selection of the more salient luminance feature. In any case, the effect of such a hierarchical selection mechanism is to improve the efficiency of multidimensional target selection by restricting higher order processing to a subset of the available stimuli and thereby selectively enhance the neural signal of the stimuli having all of the relevant features/attributes.

The present results are also in line with those of Boehler et al. (2011), which showed that attention to one feature of an object resulted in enhanced processing of its irrelevant feature, even when that feature was presented within a different object (in the opposite visual field). The Boehler et al. study used transient stimuli, and analysis of the ERPs showed that the spatially global facilitation of the irrelevant feature occurred about 80 ms after attention became focused on the attended object, as indexed by the N2pc component. This facilitation of the irrelevant feature appeared to be hierarchically contingent upon selection of the object, and, in accordance with the present study, this contingent effect was maximally evident at lateral posterior electrode sites.

Hierarchical allocation of attention during late stages of selection has been previously linked to stimulus competition (White, Rolfs, & Carrasco, 2015) or decisional processing (Andersen et al., 2011). However, neither explanation is readily applied to sustained surface‐based attention. One possibility is that the observed hierarchical pattern represents recurrent binding of attended features rather than their selection. Most of the previous studies of attention to feature conjunctions used basic features encodable at the level of individual neurons in early visual cortex such as color, motion, or orientation. In contrast, surface‐based selection requires dynamic updating of the link between spatial position and direction of motion of to‐be‐attended dots (Stoner & Blanc, 2010). It is possible that, in order to maintain the selection of the relevant stimulus in the present study, binding of surface‐defining features and luminance values occurred in parallel with selection, which was reflected in the differing activity of the two electrode clusters. This proposed mechanism is broadly consistent with the theory of incremental grouping (Roelfsema, 2006; Roelfsema, Lamme, & Spekreijse, 2000), which describes recurrent processing resulting in the enhancement of the responses of neural populations encoding the features to be grouped.

The approach utilized here for separating electrode clusters in the analysis of SSVEPs was initially developed by Andersen, Müller, & Martinovic (2012). The electrode clusters identified in that study were highly similar to the ones in the present study and were interpreted to most likely reflect activity in the early visual areas V1‐V3 (midline central cluster) and MT (lateral parieto‐occipital cluster). Interestingly, Andersen et al. (2012) also found qualitatively different attention effects in these clusters, with the midline central cluster exhibiting a pure attentional sensory gain modulation and the lateral parieto‐occipital cluster showing a pattern more consistent with the joint operation of sensory gain and competitive stimulus interactions (Bundesen, 1990; Desimone & Duncan, 1995; Reynolds & Heeger, 2009). Taken together, the present study and that of Andersen et al. (2012) demonstrate that scalp‐recorded SSVEPs can be readily separated into different components, providing windows into the operation of functionally distinct steps of visual analysis (see also Ales & Norcia, 2009; Cottereau, Ales, & Norcia, 2012).

It is important to note that the present task could produce accurate performance only if selection was done using the combination of luminance polarity and direction of motion. Since the stimuli were fully overlapped, top‐down spatial selection could not be used. Due to the configuration of the stimuli selection by luminance polarity, direction of motion or depth alone was also not possible. While a specific flicker frequency was linked to each individual group of dots, previous studies have ruled out flicker frequency as a useful cue for selection in such multidimensional attention studies using behavioral control experiments (Müller et al., 2006; Stormer, Winther, Li, & Andersen, 2013).

To conclude, this study showed that surface‐based attentional selection is implemented in a manner that resembles the selection of other types of features/attributes. When subjects attended to a conjunction of a given surface and luminance level, we found evidence that attention produced an independent multiplicative enhancement of the neural activity elicited by dots on the attended surface and dots of the attended luminance. Importantly, the luminance‐based selection was not restricted by the surface boundaries. These independent and parallel selections of surface and luminance took place in neural pathways that produced maximal SSVEP amplitudes at midline occipital electrode sites. In contrast, the SSVEPs elicited at more lateral parieto‐occipital electrodes showed a hierarchical pattern of selection, with enhancement of the neural activity elicited by the attended surface contingent upon its having the attended luminance value. This pattern suggests that the neural pathways producing the more lateral SSVEPs were integrating the surface and feature attributes of the attended conjunction, most likely at a higher processing level than the midline pathways. One possibility is that these laterally recorded pathways are engaged in a feature‐binding operation, whereby the surface and luminance representations are combined. Such binding would most likely involve complex mechanisms ensuring continuous and dynamic updating of feature‐surface conjunctions. Thus, while attending to a moving surface requires more elaborate neural computations than attending to a simple feature such as color or luminance, at least in some respects surface selection conforms to the same rules as selection of more simple features.

## AUTHOR CONTRIBUTIONS

S.K.A. and S.A.H. designed research; S.K.A. performed research; N.A. and S.K.A. analyzed data, N.A. wrote the first manuscript draft and all authors revised the manuscript.
